# Improved Process for the Continuous Acylation of 1,3-Benzodioxole

**DOI:** 10.3390/molecules29030726

**Published:** 2024-02-04

**Authors:** Davide Pollon, Francesca Annunziata, Stefano Paganelli, Lucia Tamborini, Andrea Pinto, Sabrina Fabris, Maria Antonietta Baldo, Oreste Piccolo

**Affiliations:** 1 Dipartimento di Scienze Molecolari e Nanosistemi, Università Ca’ Foscari Venezia, Via Torino 155, Venezia Mestre, 30170 Venezia, Italy; pollon.davide@gmail.com (D.P.); spag@unive.it (S.P.); sabri@unive.it (S.F.); toni@unive.it (M.A.B.); 2 Dipartimento di Scienze Farmaceutiche, Università degli Studi di Milano, Via Mangiagalli 25, 20133 Milano, Italy; francesca.annunziata@unimi.it (F.A.); lucia.tamborini@unimi.it (L.T.); 3 Dipartimento di Scienze per gli Alimenti, la Nutrizione e l’Ambiente, Università degli Studi di Milano, Via Celoria 2, 20133 Milano, Italy; andrea.pinto@unimi.it; 4 Consorzio Interuniversitario Reattività Chimica e Catalisi (CIRCC), Via Celso Ulpiani 27, 70126 Bari, Italy; 5 Studio di Consulenza Scientifica SCSOP, Via Bornò 5, 23896 Sirtori, Italy

**Keywords:** Brønsted or Lewis acids, heterogeneous recyclable catalyst, acylation of 1,3-benzodioxole, flow chemistry

## Abstract

The acylation of 1,3-benzodioxole was studied in a continuous process using a recyclable heterogeneous substoichiometric catalyst. In a short time period (30 min), at 100 °C, the conversion rate was 73%, with a selectivity of 62% of the desired acylated product; the reaction was run continuously for 6 h, showing excellent stability and selectivity. Moreover, the unreacted starting material, 1,3-benzodioxole, can be easily separated by distillation and recycled.

## 1. Introduction

**Scheme 1:**
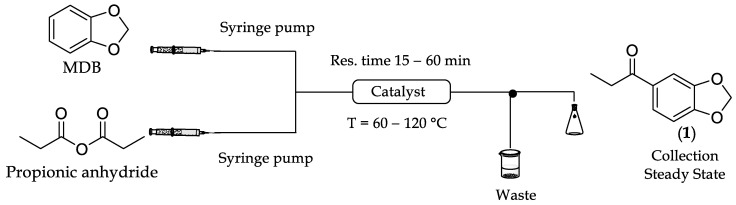
A schematic representation of the flow set up for the synthesis of compound (**1**).

Friedel–Crafts acylation and alkylation reactions are still widely exploited nowadays to introduce an acyl or an alkyl group during the synthesis of bioactive molecules, natural products, cosmetics, pharmaceuticals, and agrochemicals [1,2]. Although significant progress has been made in improving the environmental friendliness of this reaction, traditionally harsh conditions (e.g., excess AlCl_3_, ether, or chlorinated solvents) are still routinely employed. Therefore, many efforts have been made to increase the efficiency, sustainability, and scalability of this reaction. A promising approach is the development of continuous flow Friedel–Crafts reactions using both homogeneous and heterogeneous catalysts. The potential advantages of a continuous flow approach include improved heat and mass transfer, precise temperature control, easier scale-up, the generation of less waste, and the integration of reaction monitoring and processing steps. Moreover, the use of heterogeneous catalysts in packed bed reactors (PBRs) is particularly advantageous because it can obviate the need for both the separation of catalysts and workup procedures, reducing manual handling and time. Furthermore, the high local concentration of the catalyst allows the achievement of faster kinetics compared to the batch ones [3,4,5]. Pioneering work on continuous-flow Friedel–Crafts reaction using a heterogeneous catalyst was achieved by Poliakoff and coworkers [6]. They exploited sulfonic acid supported on polysiloxane (Deloxane^®^) to catalyze the alkylation of mesitylene and anisole with propene or propane-2-ol in supercritical propene or CO_2_ as a solvent under continuous-flow conditions. However, only low levels of chemoselectivity were obtained, and harsh conditions (160 °C and 200 bar) were necessary. Other heterogeneous catalysts, such as Fe_2_O_3_ nanoparticles supported on Al-SBA-15 [7] or Nafion-H [8], have also been used for flow Friedel–Crafts-type reactions under high-temperature and pressure conditions. In this context, we became intrigued by the acylation of 1,3-benzodioxole (MDB), a very important industrial intermediate for the synthesis of the insecticide synergist 5-{[2-(2-butoxyethoxy) ethoxy] methyl}-6-propyl-2*H*-1,3-benzodioxole (PBO) [9], of the important fragrance 3-(1,3-benzodioxol-5-yl)-2-methylpropanal, Helional^®^ [10,11], and of many other active ingredients. In this work, the acylation reaction of MDB to selectively produce the valuable compound 1-(benzo[*d*][1,3]dioxol-5-yl)propan-1-one (**1**) has been studied under flow conditions and exploits a recyclable heterogeneous acidic catalyst, as illustrated in Scheme 1.

For the industrial production of 1-(benzo[*d*][1,3]dioxol-5-yl)propan-1-one (**1**), by using propionic anhydride as an acylating agent, different catalysts based on Brønsted acids, such as methane sulfonic acid [12] or trifluoroacetic acid [13], Lewis acids, such as zinc chloride [14], and a mixture composed of zinc chloride and zinc oxide [15] have already been patented or published in the literature. However, in these ways, a large amount of waste is also usually generated. Some heterogeneous catalysts were also described for Friedel–Crafts acylation [15,16,17] in batch mode or in a continuous process, but, to the best of our knowledge, the continuous acylation of 1,3-benzodioxole was not studied in detail.

In the last few years, our group has proposed commercial or easily preparable heterogeneous recyclable catalysts either for the acylation of aromatic substrates or as greener Brønsted or Lewis acids and conducted the reactions in a batch mode or by a continuous process [18,19]. In particular, AquivionSO_3_H^®^ and its salts have been considered. In the case of electron-rich substrates, for example, thiophene derivatives, a Lewis catalyst such as Fe-Aquivion was used in a very small amount and has been efficiently recycled twice [18]. In this case, 2-ethylthiophene was converted into 2-acyl-5-ethyl-thiophene, a substance with an anise-like odor, in a practically complete conversion and selectivity [18]. Following these previous investigations and promising results, here, we decided to explore the performance of the acylation reaction of 1,3-benzodioxole conducted in the presence of these heterogeneous catalysts.

**Figure 1:**
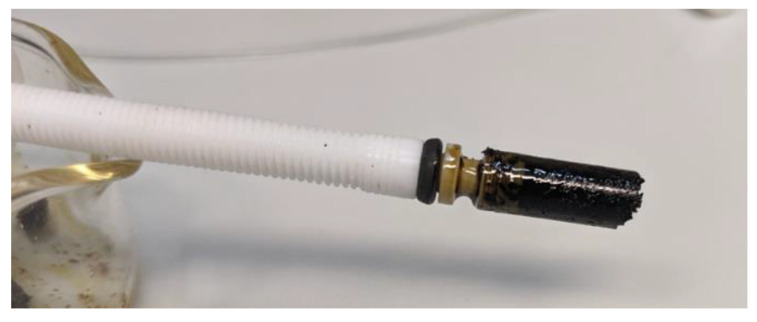
Picture of Aquivion SO_3_H^®^ after its use under continuous flow conditions at 140 °C.

It is worthy to point out that the use of AquivionSO_3_H^®^ and/or its salts as catalysts was suggested by us to a producer of 1-(benzo[*d*][1,3]dioxol-5-yl)propan-1-one (**1**), who submitted a patent application independently from us [20]. Moreover, very recently, some of the inventors of that patent described their work on this reaction performed under a batch mode [21]. Our objective, instead, was to investigate the production process of compound (**1**) by working under a continuous mode. It is also worth noting that in the patent application [20], a few experiments on flow were described, but in our hands, the results were not reproducible because the catalyst, under the described conditions, was completely destroyed, as shown in Figure 1. To verify the consistency of this issue, the procedure was performed twice by using a new catalyst. In any case, AquivionSO_3_H^®^ at 140 °C formed a compact block that clogged the reactor, causing the pumps to stop due to overpressure (>10 bar). Therefore, on the basis of our tests, a temperature of 120 °C has been considered as the limit for this catalyst. In the present work, the continuous acylation reaction of MDB was studied under different conditions of stoichiometry, temperature, and residence time, and the reaction outcome was evaluated by gas chromatographic analysis.

## 2. Results and Discussion

First, the acylation reaction of 1,3-benzodioxole was performed in batches by following the procedure described in Materials and Methods Section. In all the reaction conditions adopted, the level of selectivity was never very high due to the formation of by-products. Concerning the metal salts, the use of Zn-Aquivion (Zn-Aq) gave the best results. As a matter of fact, in the presence of Zn-Aq, the best conversion was 59%, but the level of selectivity to the acylated product was only 34%. Selectivity increased to 41% by limiting the conversion to 31%.

With the aim of increasing both conversion and selectivity, silica-bonded sulfonic acid, as an alternative to AquivionSO_3_H^®^, was tested in batch mode. Preliminary results showed that this catalyst was less active than AquivionSO_3_H^®^, but the data obtained appeared quite promising, and silica-bonded sulfonic acid could represent a valid alternative if Aquivion were removed from the market in the future.

Then, we decided to study the stability and reactivity of MDB in the presence of Zn-Aquivion and propionic anhydride or propanoyl chloride, performing the reaction in batch at different temperatures; the results obtained are collected in Table 1. Using propionic anhydride, the reaction is not occurring at room temperature; neither by-products are formed after 24 h, while at 50 °C and 75 °C, no product was detected, but the formation of some by-products was observed after 60 min. On the contrary, using propanoyl chloride at 75 °C, it was possible to detect the desired product. However, the amount of by-products was higher than using propionic anhydride, and, therefore, we decided to continue our investigation using propionic anhydride and higher temperatures.

Then, the stability of product (**1**) in the presence of dry glacial acetic acid and a mixture of glacial acetic acid and water (1% *v*/*v*) at different temperatures was also evaluated. From the results obtained (see Table 2), it was observed that compound (**1**) was stable in all the tested conditions.

**Figure 2:**
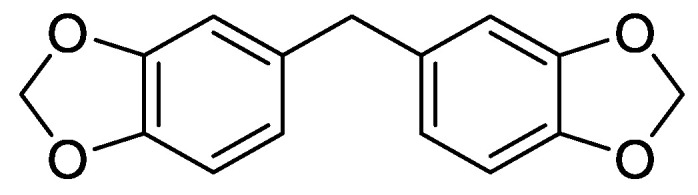
The structure of bis(benzo[*d*][1,3]dioxol-5-yl)methane formed in the presence of MDB and Zn-Aquivion at 120 °C after a residence time of 15 min.

To assess the behavior in the flow system of the chemical species involved (MDB, propionic anhydride, and final product (**1**)), stability experiments under continuous conditions were then performed. To this aim, a column was packed with a mixed bed of Zn-Aquivion (0.25 g) and silica (0.25 g), and the reagents as such or in solution (dichloroethane) were flown through the packed bed reactor with a residence time of 15 min at 120 °C. The results can be summarized as follows:

- Compound (**1**) is stable;
- Compound (**1**) in the presence of glacial acetic acid is stable;
- Propionic anhydride is stable;
- Compound (**1**) (a 20 mg/mL solution in dichloroethane) is stable;
- MDB forms a less polar by-product in contact with the catalyst at 120 °C whose structure, according to NMR data (^1^H NMR (300 MHz, CDCl_3_) δ 6.78–6.55 (m, 6H), 5.94–5.88 (m, 4H), 3.80 (s, 2H), should be bis(benzo[*d*][1,3]dioxol-5-yl)methane (Figure 2). The isolated yield was <5%.

The reaction was then studied under flow conditions using Zn-Aquivion or AquivionSO_3_H^®^ as catalysts (Scheme 1). The catalyst was packed in a glass column as such or mixed with silica, and neat MDB and propionic anhydride were flowed through it after mixing in a T-piece. No swelling of the catalyst was observed during the reactions. Different parameters (i.e., residence time: 15, 30, and 60 min; stoichiometry: MDB/propionic anhydride 1:2, 2:1, and 1:1; and temperature: 80 °C, 100 °C, and 120 °C) were varied, and the reaction outcome was evaluated by GC collecting samples at the steady state.

In detail, gas chromatographic characterizations of the reagent MDB and product (**1**) were preliminarily performed by injecting one by one standard acetonitrile solutions containing 20 mg L^−1^ of each compound. Under the optimized experimental operating conditions employed here, well-defined and resolved peaks for both reagent MDB and product (**1**) were obtained, characterized by the retention times shown above (see the Section 3.2, Figure 4). For the quantitative evaluation of the reaction outcome studied under the different conditions employed here, calibration plots for reagent MDB and product (**1**) were performed by analyzing standard solutions containing both compounds in the concentration range 10–100 mg L^−1^ and mesitylene (Mes) at a constant concentration of 25 mg L^−1^. Linear calibration plots were obtained in both cases, with the following linear regression equations and correlation coefficients (R^2^): A_MDB_/A_Mes_ = 0.0255 C_MDB_ (mg L^−1^) + 0.1058, R^2^ = 0.9992, A_(**1**)/_A_Mes_ = 0.0239 C_(1)_ (mg L^−1^) + 0.2051, and R^2^ = 0.9989, where A_MDB_, A_(1)_, and A_Mes_ are chromatographic peak area values of MDB, product (**1**), and mesitylene, and C_MDB_ and C_(1)_ are the standard concentrations of compounds, respectively.

**Figure 3:**
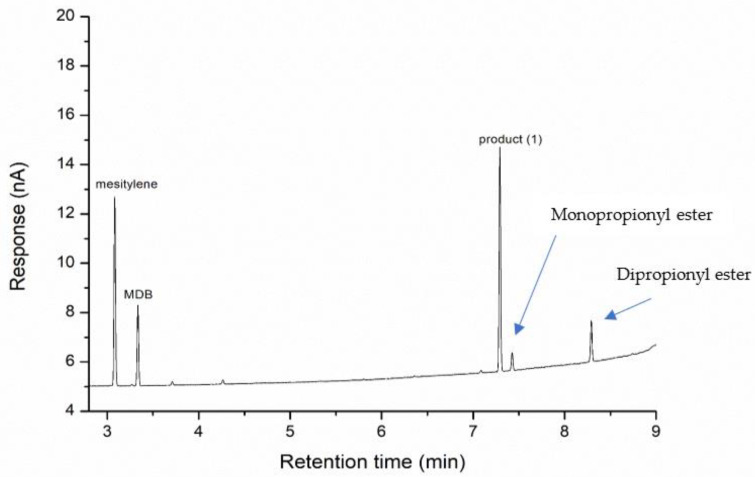
GC chromatogram of a reaction crude. Reaction conditions: propionic anhydride 1:1 and Aquivion SO_3_H^®^, temperature reaction 100 °C, residence time 30 min; internal standard mesitylene, 25 mg L^−1^.

The outcome of the reaction was then evaluated by analyzing, in duplicate, all the samples prepared by the flow synthesis of the compound (**1**) conducted under the different experimental conditions tested here. Figure 3 shows, for instance, the GC response obtained for a sample obtained by the reaction conducted in a molar ratio of MDB/propionic anhydride 1:1 with the AquivionSO_3_H^®^ catalyst, with a temperature reaction of 100 °C and a residence time of 30 min, then suitably dissolved in acetonitrile and added with mesitylene. As can be observed, peaks at retention times corresponding to the reagents MDB, product **1**, and mesitylene are identified. Moreover, in this case, two small peaks at higher retention times than product (**1**), i.e., at about 7.43 min and 8.30 min, and the other two very little peaks just emerging from the background, are evidenced, probably due to some by-products formed during the reaction. On the basis of HPLC, GC, and GC-MS characterizations, we tentatively defined the structure of the two main impurities of this reaction, which were conceivably assigned to mono and di propionyl derivates of catechol, and we think that their formation is due to the water formed during the reforming of propionyl anhydride from propionic acid. In Figure 3, the gas chromatogram of the reaction mixture is reported.

To evaluate the conversion of MDB and the calculated yield of compound (**1**) obtained by the different synthesis procedures, the quantitative determination of the content of both MDB and product (**1**) in each sample was performed by interpolating the experimental values of their chromatographic peak areas, normalized by the relevant internal standard area, with the corresponding linear regression equation. The results we found are summarized in Table 3.

As reported in Table 3, the Brønsted catalyst is more active even if the Aquivion salt is more stable and could be used at a higher temperature. Runs 5–7 show that the unmodified AquivionSO_3_H^®^ is stable, and reproducible results are obtained (similar to runs 8–9). The best results were obtained within 30 min of residence time at 100 °C using AquivionSO_3_H^®^ as catalyst and with a 1:1 ratio of MDB/propionic anhydride (runs 6 and 7). In fact, exploiting these conditions, conversion values of 71–73% were achieved, and the calculated yield was about 61–62%. The reactor was run for 6 h, flowing 7.0 mL of MDB. At the end, a sample was collected and analyzed by GC, showing the same conversion and selectivity. The desired product (**1**), after purification, was isolated with a 60% yield.

## 3. Methodology

### 3.1. Materials and Methods

A sample of AquivionSO_3_H^®^ was kindly provided by Dr. Oldani (Solvay, Italy) and used as such. Other reagents and solvents were of high purity, purchased from Merck, Fluka, and AlfaAesar, and used without any further purifications. If not specified, the solvents used in this work were of HPLC grade (purchased from Merck, Fluka, and AlfaAesar). GC–MS analyses were performed on a HP 5890 series II gas chromatograph interfaced to a HP 5971 quadrupole mass detector.

Continuous flow reactions were performed using Asia syringe pumps from Syrris (Asahi Glassplant UK Ltd., Royston, UK) coupled with a R4 heating block from Vapourtec equipped with an Omnifit^®^ glass column (6.6 mm i.d. × 10 mm length). The temperature sensor sat on the walls of the reactors. ^1^H NMR was recorded in CDCl_3_ on a Varian Mercury at 300 MHz. Chemical shifts (in ppm) were referred to the residual hydrogen/carbon solvent peaks. HPLC analyses were performed using a Jasco PU-980 pump, exploiting ChromNAV 2.0 software, and a Phenomenex Luna 5u Silica (2) 100 Å, 250 mm × 4.6 mm (eluent: hexane/*i*PrOH 9:1; flow rate: 0.8 mL min^−1^). A Jasco UV-975 Intelligent UV/VIS Detector was used (λ = 210 nm).

### 3.2. Gas Chromatographic Experiments

Gas chromatographic (GC) measurements were performed using an Agilent model 8860 GC system (Agilent Technologies Italia Spa, Milan, Italy) equipped with a flame ionization detector (FID) and a split/splitless injector, set in split mode. The column used was a capillary column Agilent HP-5, 5% phenyl methyl silicon (30 m × 0.32 mm i.d.; film thickness: 0.25 μm), employing pure N_2_ (99.99%, SIAD, Bergamo, Italy) as the mobile phase. For the burner flame, pure H_2_ (99.99%) and air (99.99%) were also supplied by SIAD. The GC operation and chromatographic outputs were managed by Agilent “OpenLab CDS 2.8” software.

The parameters of the chromatographic analysis were chosen after an optimization step to find the best conditions in terms of their peak resolution, sensitivity, and analysis time. The selected operating conditions and modes were the following: The temperature of the injector was set at 250 °C and that of the detector at 290 °C; the carrier gas in the column and the split gas (both N_2_) were fixed at constant flow rates of 1.5 and 90 mL min^−1^, respectively, thus a split ratio of 60:1; during measurements, the FID burner was powered by H_2_ and air at flow rates of 30 and 400 mL min^−1^, respectively, and kept under a constant flow rate of N_2_, as a make-up inert gas, of 25 mL min^−1^. Regarding oven temperature (T) programming, the following optimized conditions were chosen: initial T column: 100 °C, initial time: 2 min; T ramp: 20 °C min^−1^; final T column: 250 °C; and post-run T: 280 °C, time 2 min.

**Figure 4:**
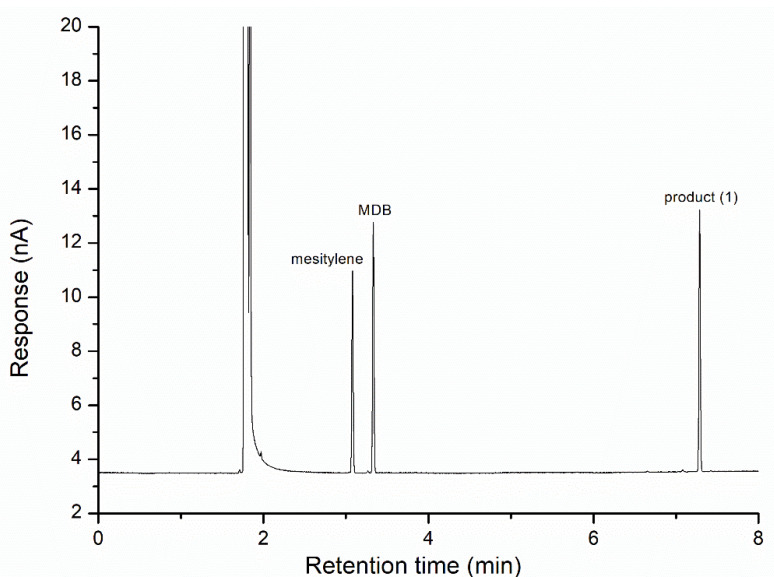
Identification of the retention times of MDB and product (**1**), 1-(benzo[*d*][1,3]dioxol-5-yl)propan-1-one.

Under such conditions, the total elution time of each chromatographic run was about 7.3 min. Figure 4 shows, as an example, a typical GC response recorded in a acetonitrile standard solution containing MDB and product (**1**), 1-(benzo[*d*][1,3]dioxol-5-yl)propan-1-one, both 40 mg L−1, and the internal standard mesitylene, 25 mg L−1. From such chromatographic runs, the following retention times of the analytes were obtained: MDB, 3.332 ± 0.006 min; 1-(benzo[*d*][1,3]dioxol-5-yl)propan-1-one, 7.288 ± 0.004 min; and mesitylene, 3.079 ± 0.005 min.

For sample micro-injections, glass Hamilton microliter syringes, 701 Series, 10 μL (Hamilton Italia S.r.l., Agrate Brianza, Italy), were employed. For each measurement, a sample volume of about 1.0–1.2 μL was withdrawn and injected.

For the quantification of analytes, the calibration plot with the internal standard method was applied using mesitylene (1,2,5 trimethylbenzene), a pure analytical standard (Merck, Darmstadt, Germany), as an internal standard compound. Acetonitrile, HPLC grade, purity ≥ 99.9% from Sigma-Aldrich-Merck, was used as a solvent to prepare the standard and sample solutions, as well as the blank solution. To construct the calibration plots for both reagents, MDB, and the product of the reaction under investigation, standard solutions of each compound were prepared by dilution in acetonitrile to obtain a set of six diluted solutions in the concentration range of 10–100 mg L^−1^ and adding mesitylene at a constant concentration of 25 mg L^−1^. Each standard, as well as the sample solutions, was analyzed in duplicate.

To identify the main by-products, an HPLC/mass GC Agilent 8860 instrument coupled to a mass Agilent 5977 GC/MSD (Santa Clara, CA, USA) single quadrupole was used, equipped with a column HP5Agilent J&W (30 m; internal diameter: 0.32 mm; film thickness: 0.25 μm). The analysis conditions were set as follows: temperature injector at 250 °C: split mode (split ratio: 60:1) pressure: 16,191 psi; split flow: 90 mL/min; gas saver: 20 mL/min after 2 min; and injected sample volume: 1 μL.

The oven temperature was programmed with an initial temperature of 100 °C for 2 min; T ramp 20 °C/min up to 250 °C; the final temperature of 250 °C was maintained for 5 min. Transfer line temperature (oven-source): 250 °C; the mass source temperature was set at 250 °C, and the quadrupole temperature was set at 150 °C. Acquisition was conducted in SCAN mode, with a filtered mass range of 50–500.

First, a sample of the reaction mixture was washed with an alkaline solution, and the separated aqueous phase was analyzed after neutralization. The retention time of compound (**1**) was 7.38 min.

Mass spectrum of by-product A (retention time: 6.53 min): *m*/*z* = 166 [M]^+^(16), 110 (68), and 57 (100).

Mass spectrum of by-product B (retention time: 7.41 min): *m*/*z* = 222 [M]^+^, 166 (21), 110 (63), and 57 (100).

The mass spectrum of catechol that was formed by the treatment with an alkaline solution was well compared with a known substrate. It should be possible to eliminate these by-products with an aqueous alkaline solution in a more accurate condition.

### 3.3. Description of the Procedure to Prepare Salts of Aquivion

#### Synthesis of the Zn-Aq Catalyst

A total of 10 g of Aquivion-H 87-S, 3.8 mmol of zinc, and 50 mL of acetonitrile were introduced into a 250-mL double-necked flask equipped with a reflux condenser and a mechanical stirrer. The mixture was heated under reflux for 48 h; then, the catalyst was filtered, washed with acetonitrile, and dried under vacuum. It appeared as a coarse white powder and was stored in a container under an inert atmosphere.

Using a similar procedure, the corresponding Fe(III) and Cu (II) catalysts, as well as other salts, such as aluminum and tin compounds, were prepared.

### 3.4. Synthesis of 1-(benzo[d][1,3]dioxol-5-yl)propan-1-one (***1***) Using MeAq (Me = Fe, Zn, Cu, Al, and Sn)

A total of 2 g (0.016 mol) of 1,3-benzodioxole, 0.019 mol of propionic anhydride, and approximately 0.14 g of MeAq (ratio molar catalyst substrate of 100 to 1) were used for this procedure. The mixture was heated to 160 °C and kept under stirring for 1 h (sampling after 30 min and at the end of the procedure); then, the raw mixture was cooled and filtered on a Gooch filter to recover the catalyst and to be able to carry out one test of recycling the catalyst with fresh substrates.

### 3.5. Synthesis of 1-(benzo[d][1,3]dioxol-5-yl)propan-1-one (***1***) Using Aquivion SO_3_H^®^

A total of 2 g (0.016 mol) of 1,3-benzodioxole, 0.019 mol of propionic anhydride, and approximately 0.14 g of Aquivion SO_3_H^®^ (catalyst substrate molar ratio of 100 to 1) were used for this procedure. The mixture was heated up to 120 °C and kept under stirring for 1 h (sampling after 30 min and at the end of the procedure); then, the raw mixture was cooled and filtered on a Gooch filter to recover the catalyst and to be able to carry out one test of recycling the catalyst with fresh substrates.

### 3.6. General Procedure for the Flow Synthesis of 1-(benzo[d][1,3]dioxol-5-yl)propan-1-one (***1***)

1,3-Benzodioxole and propionic anhydride were flown through the packed bed reactor by syringe pumps. The molar ratio between the reagents was controlled by the flow rate as well as the residence time. The samples were always collected at the steady state (after 2 column volumes). The reactor was packed with pure catalyst (0.50 g) or mixed with silica (0.25 g of silica and 0.25 g of catalyst).

### 3.7. Protocol for Molar Ratio 1:1—Residence Time 15 Min

A glass column (6.6 mm i.d.) was packed with 0.25 g of Zn-Aquivion catalyst and 0.25 g of silica (final volume: 0.62 mL). 1,3-Benzodioxole (19.5 µLmin^−1^) and propionic anhydride (21.8 µLmin^−1^) were flown individually by syringe pumps with a total residence time of 15 min. The temperature was set at 80 °C, 100 °C, and 120 °C and controlled by a temperature sensor sitting on the wall of the column. The samples were collected at the steady state. To obtain the pure final compound (**1**), the crude was purified through a chromatographic column on silica, using as the mobile phase the mixture cyclohexane: ethyl acetate = 95:5. The isolated product was characterized by ^1^H NMR and GC–MS.

^1^H NMR (300 MHz, CDCl_3_) δ 7.56 (dd, *J* = 8.2, 1.7 Hz, 1H), 7.44 (d, *J* = 1.7 Hz, 1H), 6.84 (d, *J* = 8.2 Hz, 1H), 6.03 (s, 2H), 2.92 (q, *J* = 7.3 Hz, 2H), and 1.20 (t, *J* = 7.3 Hz, 3H). GC-MS (*m*/*z*): 178[M^+^] (42), 149[M-C_2_H_5_]^+^ (100), and 121[M- C_2_H_5_-CO]^+^ (47).

The flow rates were modulated to modify the stoichiometry (1:1, 2:1, and 1:2) and the residence time (15, 30, and 60 min).

## 4. Conclusions

In summary, in this paper, a sustainable and straightforward continuous process for the acylation of 1,3-benzodioxole (MBD) in the presence of easily recyclable heterogeneous catalysts such as Zn-Aquivion or AquivionSO_3_H^®^ alone has been reported. The procedure described, developed by us, allows for the obtainment of a key compound in the synthesis of the fragrance Helional^®^ and of many other active ingredients. Our results show that it is possible to obtain 1-(benzo[*d*][1,3]dioxol-5-yl)propan-1-one (**1**) in a short time with fine/excellent conversion and selectivity. Under optimized flow conditions, using 0.5 g of catalyst (volume of the packed bed reactor = 0.62 mL), the productivity was 1.08 g_pruduct_/h (13.0 g_product_/g_catalyst_). By comparison with the batch synthetic procedure, higher substrate conversions, lower reaction times, and increased selectivity for the acylated product (**1**) were obtained, making the continuous process more convenient and sustainable. The obtained results are comparable to or better than those recently published [20,21] for this reaction and, in principle, more suitable for industrial scaling-up.

## Figures and Tables

**Table 1:** Evaluation of the stability and reactivity of MDB in presence of Zn-Aquivion and propionic anhydride or propanoyl chloride in batch ^a^.

Compound	Reagent	Temperature	Result
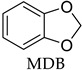	Propionic anhydride + Zn-Aquivion	r.t.	No reaction
		50 °C	By-products
		75 °C	By-products
	Propanoyl chloride + Zn-Aquivion	r.t.	No reaction
		50 °C	By-products
		75 °C	By-products + product

^a^ Conditions: a mixture of MBD (500 uL, 1 eq), propanoyl chloride (387 uL, 1 eq), or propionic anhydride (504 uL, 1 eq) and Zn-Aquivion (15 mg) was stirred for 24 h at the appropriate temperature.

**Table 2:** Evaluation of the stability of compound (**1**) in batch in the presence of dry glacial acetic acid and a mixture of glacial acetic acid and water (1% *v*/*v*).

Compound	Reagents	Temperature	Result
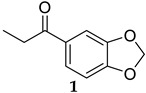	Glacial acetic acid (dry)	Reflux	Stable
	Acetic acid + 1% of H_2_O	Reflux	Stable
	Dichloroethane + Zn Aquivion	r.t.	Stable
		50 °C	Stable
		75 °C	Stable
	Propionic anhydride + Zn Aquivion	r.t.	Stable
		50 °C	Stable
		75 °C	Stable

**Table 3:** Experimental data found from the continuous acylation reaction of MDB with propionic anhydride, conducted under different conditions of stoichiometry, temperature, and residence time.

Run	Catalyst	Ratio of MDB/Propionic Anhydride	Reaction Conditions (Temperature and Residence Time)	Conversion of MDB %	Calculated Yield of (1) %
1	Zn-Aquivion + Silica	1/2	120 °C and 15 min	70	22
2	Zn-Aquivion + Silica	2/1	120 °C and 15 min	27	20
3	AquivionSO_3_H^®^	1/1	100 °C and 15 min	72	39
4	AquivionSO_3_H^®^	1/1	100 °C and 15 min	71	40
5	AquivionSO_3_H^®^	1/1	100 °C and 30 min	70	59
6	AquivionSO_3_H^®^	1/1	100 °C and 30 min	73	62
7	AquivionSO_3_H^®^	1/1	100 °C and 30 min	71	61
8	AquivionSO_3_H^®^	1/1	80 °C and 15 min	61	48
9	AquivionSO_3_H^®^	1/1	80 °C and 15 min	61	47
10	AquivionSO_3_H^®^	1/1	80 °C and 60 min	67	46
11	AquivionSO_3_H^®^	1/1	80 °C and 60 min	68	48

## Data Availability

Data are contained within the article.
